# Design and torque control base on neural network PID of a variable stiffness joint for rehabilitation robot

**DOI:** 10.3389/fnbot.2022.1007324

**Published:** 2022-11-18

**Authors:** Bingshan Hu, Binghao Mao, Sheng Lu, Hongliu Yu

**Affiliations:** ^1^Institute of Rehabilitation Engineering and Technology, University of Shanghai for Science and Technology, Shanghai, China; ^2^Shanghai Engineering Research Center of Assistive Devices, Shanghai, China

**Keywords:** mechatronics, variable stiffness actuators, rehabilitation robotics, neural network PID, torque control

## Abstract

Variable stiffness joints have been gradually applied in rehabilitation robots because of their intrinsic compliance and greater ability to adjust mechanical stiffness. This paper designs a variable stiffness joint for upper limb rehabilitation training. The joint adopts the variable stiffness principle based special curved surface. The trapezoidal lead screw in the variable stiffness module has a self-locking function, and the stiffness can be maintained without the continuous output torque of the motor. In the aspect of control, back propagation (BP) neural network PID control strategy is used to control the torque of variable stiffness joint. Experiments show that this control method can effectively improve the torque control performance of variable stiffness joints in the case of low stiffness, and the isotonic centripetal resistance training can be realized by using the joints and control methods designed in this paper.

## Introduction

Based on medical theory, the upper limb rehabilitation robot drives the affected limb to carry out scientific and effective training (Xu et al., 2011), so that the patient's motor function can be recovered better (Lin et al., 2003). According to the process of patient rehabilitation, the rehabilitation training stage of upper limb rehabilitation robot includes passive training (Lizheng et al., 2015), robot-assisted training (Chang and Kim, 2013) and resistance training (Song et al., 2014). During passive rehabilitation training, the joints of rehabilitation robot are required to have high stiffness to ensure the stability and high bandwidth of closed-loop position control, so as to drive the limbs of patients to reach the specified position accurately (Gopura et al., 2016). In the stage of assisted and resistance training, the rehabilitation robot must have good flexibility and exert different forces on the patient's limbs to ensure the safety and comfort of the rehabilitation process (Marchal-Crespo and Reinkensmeyer, 2009). It can be seen that at different stages of rehabilitation training, the driving joints of the rehabilitation robot need to have different stiffness (Ma et al., 2016). The traditional robot usually collects a large number of position, torque, speed and other data by increasing the type and number of sensors on the basis of rigid driving joints (Palazzolo et al., 2019), and then designs a controller that can process these data effectively, so as to achieve the effect of controlling the impedance of the rehabilitation robot (Yuan et al., 2013). This requires that the sensors, driving and control circuits of the rehabilitation robot run fast enough, and the system needs to establish an accurate dynamic model (Cestari et al., 2014). For example, the biped robot designed by Hubicki et al. (2018) uses inertial measurement units, encoders and other sensors to collect large amounts of data, and then processes these data through algorithms to achieve stable control effects. The driving joint of the traditional rehabilitation robot is not inherently compliant and cannot store energy, which leads to its inability to absorb the energy of the instantaneous impact (Caldwell et al., 2015). In recent years, some scholars have developed a variety of rehabilitation robots with variable stiffness joints instead of rigid joints. Vanderborgh et al. summarized the variable impedance actuators, and classified it into active impedance by control, inherent compliance and damping actuators, inertial actuators, etc. based on the principle of variable stiffness and impedance (Vanderborght et al., 2013). Yi et al. designed a variable stiffness joint of exoskeleton (Yi et al., 2018). Yang et al. used a variable stiffness rehabilitation robot to perform elbow rehabilitation training for stroke patients (Yang et al., 2021). Baser and Kizilhan designed a wearable ankle exoskeleton with variable stiffness (Baser and Kizilhan, 2018).

According to the working principle, the working principle of variable stiffness actuator (VSA) can be divided into variable lever arm, special curved surface, changing the number of elastic elements and so on. The way of changing lever arm is to change the transmission ratio between load and spring according to the proportion of lever arm. It is mainly composed of load point, pivot and spring contact point. Changing any position can adjust the stiffness of the mechanism. For example, Chaichaowarat designed a variable stiffness spring mechanism, which is composed of a slider, a roller and an adjustable unsupported length leaf spring. The adjustment of the slider position changes the spring contact point, thus obtaining the variable stiffness characteristics (Chaichaowarat et al., 2021). Similar variable stiffness actuators include AWAS-I (Jafari et al., 2010), AWAS-II (Jafari et al., 2014), COMPACT-VSA (Tsagarakis et al., 2011), etc. In the variable stiffness principle of special curved surface, the variable stiffness mechanism is connected in series between the reducer and the output shaft of the joint. The stiffness control motor changes the relative position of the cam disc to control the pretension force of the spring to adjust the stiffness of the joint. For example, the FSJ joint developed by Wolf, when the joint is subjected to passive torque load or the spring preload is changed, the cam disc rotates relatively, the spring is compressed, and the stiffness of the joint changes (Wolf et al., 2016). Similar special surface variable stiffness actuators include MESTRAN (Hung Vu et al., 2011), VSM (Sun et al., 2018) and SJM-II (Park and Song, 2010). Some variable stiffness joints adjust their stiffness by changing the number of elastic elements. In the discrete variable stiffness actuator designed by Hussain, the spring set is connected in series between the drive motor and the load end. The springs are controlled by the clutch, respectively. The on-off of the clutch can be realized by changing the number of spring connections (Hussain et al., 2021). In addition, there are other methods. For example, Garabini et al. designed a soft robots that mimic the neuromusculoskeletal system, which reproduces many of the characteristics of an agonistic-antagonistic muscular pair acting on a joint (Garabini et al., 2017). For the variable lever arm type variable stiffness joint, the torque curve depends on the length of the lever. If the length of the lever is increased, the volume of the mechanism will increase accordingly. For the variable number of elastic elements type, the volume of the mechanism will also increase due to the number of elastic elements. The ideal asymptote torque curve can be obtained only by changing the contour of the special surface, which is easier to achieve miniaturization in terms of volume and weight. Therefore, the principle of variable stiffness of the special curved surface will be used in this paper.

In terms of control, variable stiffness joints operate in a large stiffness range. In the case of low stiffness, vibration is often accompanied, and its torque response performance is also affected (Albu-Schaffer et al., 2010), which reduces the safety of human-machine interaction in rehabilitation training. To solve such problems, Liu investigated a closed-loop torque controlled variable stiffness actuator (VSA) combined with a disturbance observer, and a better dynamic response with high and low stiffness was achieved (Liu et al., 2021). Albu-Schaffer proposed a general variable stiffness joint model for nonlinear control design, and then designed a simple gain scheduling state feedback controller for active vibration reduction of weak damping joints (Albu-Schaffer et al., 2010). Misgeld designed a gain scheduling torque controller to improve the human-machine interaction characteristics of the joint. The eigenvalue of the gain scheduling is determined by the zeros and poles of the multi-channel H∞-control strategy, which can perform gain-scheduled control on multiple stiffness values of the joint in discrete time (Misgeld et al., 2017). In the interactive control of the manipulator, the gradient of the gain scheduled variable hyperplane is adjusted according to the real-time identified environmental stiffness to achieve stable position and torque control in different environments (Iwasaki et al., 2002). Mengacci et al. designed an iterative learning control scheme based on torque by decoupling the motion/stiffness of the articulated soft robot and learning the expected action of the robot, and accurately controlled the position trajectory of the articulated soft robot without changing the flexibility of the articulated soft robot. In this paper, the PID control scheme based on BP neural network is adopted to improve the torque response speed of variable stiffness actuator under different stiffness conditions (Mengacci et al., 2020).

In this paper, based on the existing research, a variable stiffness joint used in upper limb rehabilitation training is designed to improve the safety and comfort of the rehabilitation process. Compared with the previous work, the contributions of this paper can be summarized as follows.

1. From the mechanical design point of view, the joint adopts the variable stiffness principle based special curved surface. The trapezoidal lead screw in the variable stiffness module has a self-locking function, and the stiffness can be maintained without the continuous output torque of the motor.
2. From the control algorithm point of view, the PID control based on BP neural network is adopted to improve the torque control response performance of the elbow joint rehabilitation robot driven by variable stiffness joints under low stiffness by adjusting the gain parameters under different stiffness.
3. Based on the principle of variable stiffness, this paper designs a variable stiffness joint for the elbow joint, and combines the PID control method based on BP neural network to build the experimental platform of the variable stiffness elbow joint rehabilitation robot, and the isotonic centripetal resistance training experiment of elbow joint is carried out to verify the effect of BP neural network PID torque control.

The rest of this paper is organized as follows: the second section introduces the mechanical design of the variable stiffness joint based on principle of special curved surface. The third section establishes the dynamic model of the joint, studies the PID control method based on BP neural network, and conducts simulation verification. The fourth section builds an experimental platform for a variable stiffness elbow joint rehabilitation robot, and takes the elbow joint isotonic centripetal resistance training as an example to verify the effect of BP neural network PID torque control. The fifth section is conclusions and discussions.

## Mechanical design of variable stiffness joint

### Mechanical structure design

**Figure 1:**
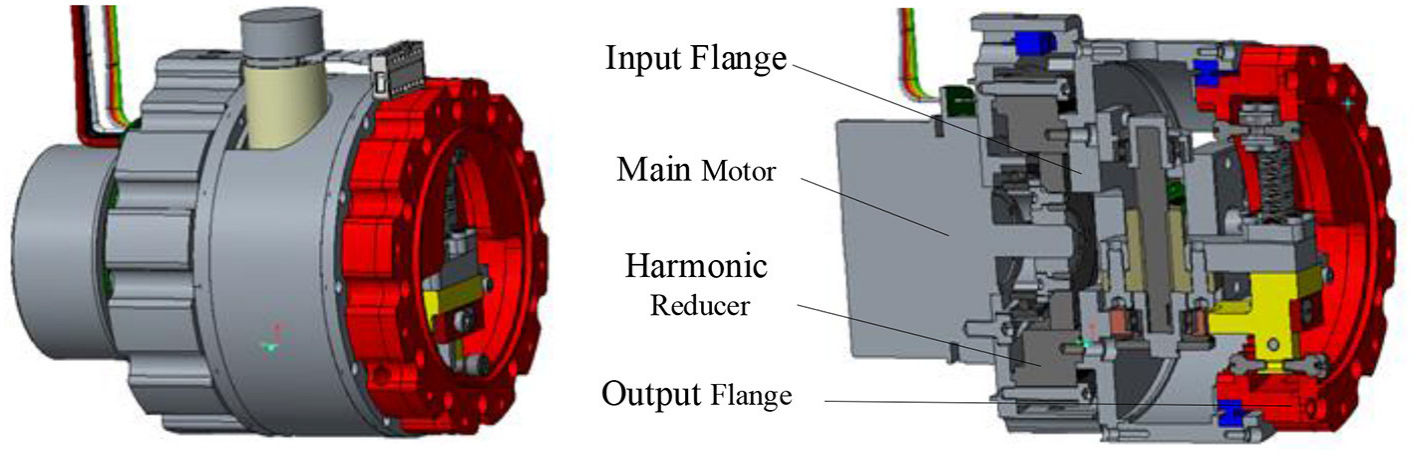
Variable stiffness joint model.

As shown in Figure 1, the overall size of the variable stiffness joint designed in this paper is 500^*^110^*^137.5mm, and the total mass is 1.5 kg. According to the structure and function, the mechanism can be divided into main drive module and variable stiffness module. The main drive module provides the output torque, and the variable stiffness module adjusts the joint output stiffness.

**Figure 2:**
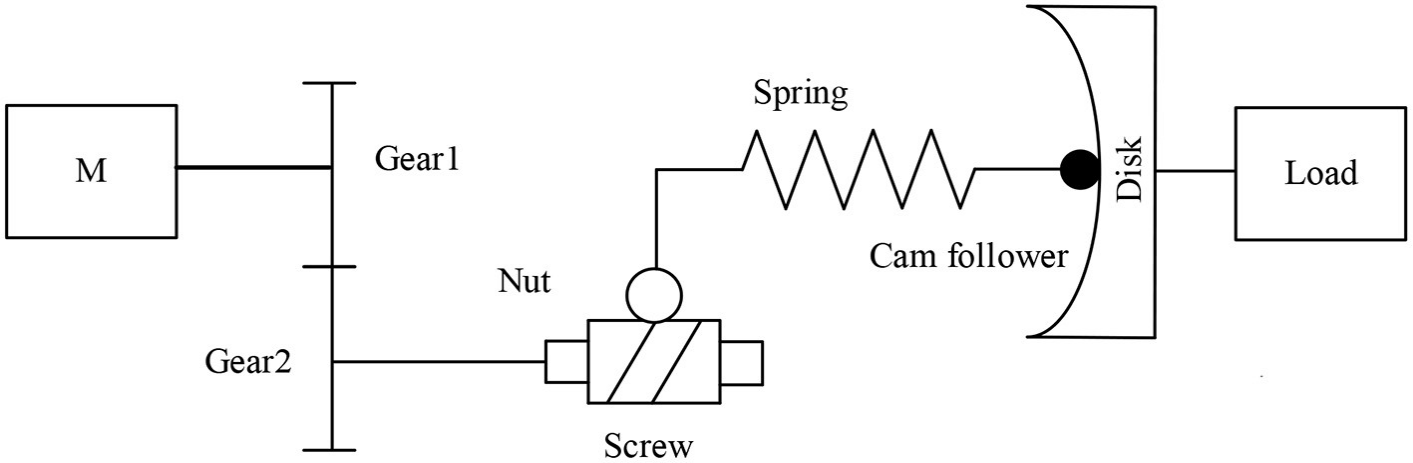
Schematic diagram of variable stiffness module mechanism.

The main drive module includes a main motor and a harmonic reducer. The main motor is a brushless DC motor with a rated torque of 269 mNm, and the reduction ratio of the harmonic reducer is 100:1. Figure 2 is the schematic diagram of the variable stiffness module mechanism. The stiffness adjustment motor drives a pair of gears to rotate, the gears drive the trapezoidal lead screw to rotate, the lead screw nut moves forward, the spring is compressed, and then the cam rotates to adjust the joint stiffness.

**Figure 3:**
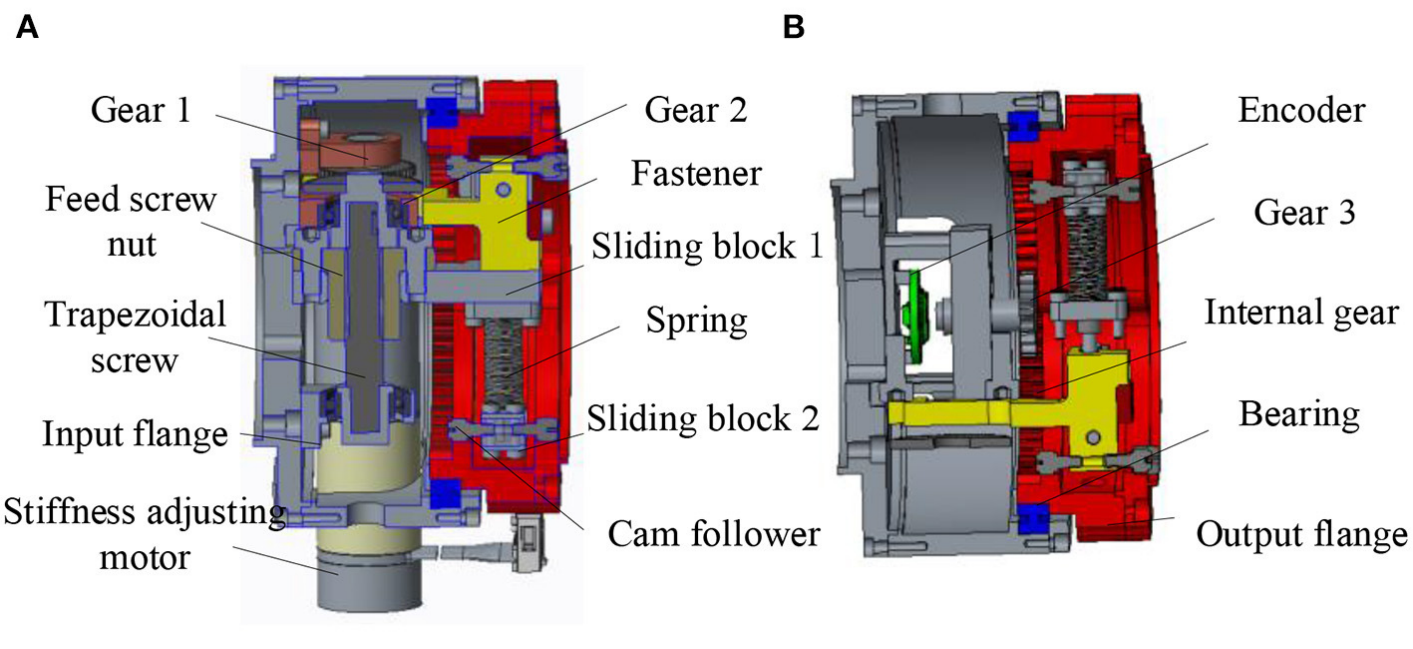
Three-dimensional model of variable stiffness joint **(A)** Sectional view 1 of variable stiffness module **(B)** Sectional view 2 of variable stiffness module.

The specific variable stiffness module is shown in Figure 3A. The left is the input flange connecting the main drive module, and the right is the output flange connecting the load. The input flange and output flange are supported by cross roller bearings. The driving source of the variable stiffness module is a brushless DC motor with a rated torque of 10.8 mNm. The output of the motor is connected with a planetary reducer. The stiffness adjustment motor is installed on the input flange. The output of the reducer drives the gear 1 to rotate, and one end of the gear 2 is meshed with the gear 1, the other end of the gear 2 is connected with the trapezoidal lead screw. The trapezoidal screw rotates to push the screw nut forward. Due to the introduction of trapezoidal lead screw, the variable stiffness module has self-locking function. So, the stiffness adjustment motor does not need to output torque while maintaining the compression of spring to reduce energy consumption. The slide block 1 installed on the right side of the lead screw nut moves forward and compresses the upper end of the spring. The lower end of the spring is connected with the sliding block 2, and a cam follower is installed on the sliding block 2. The cam follower is tangent to the cam contour in the output flange and transmits the spring force to the output flange.

In addition, as shown in Figure 3B, a rotary encoder is installed on the input flange, and the magnetic steel of the encoder is installed in the gear 3. An internal gear groove is designed in the output flange to mesh with gear 3. When there is relative rotation between the input flange and the output flange, the gear 3 rotates, and the encoder collects the rotation degree of the magnetic steel to obtain the relative rotation angle between the two flanges, that is, the deformation angle.

### Analysis of variable stiffness characteristics of special curved surface

In the variable stiffness joint with special curved surface, the design of cam contour determines the stiffness characteristics of the compliant joint, which needs to be analyzed and designed according to the contour. The variable stiffness joint designed in this paper is intended to be applied to the elbow rehabilitation robot. According to the existing literature research, the stiffness of the human elbow joint varies from 0 to 20 Nm/rad, and the output torque is 10 Nm. Therefore, the variable stiffness joint designed in this paper should meet the above index requirements.

**Figure 4:**
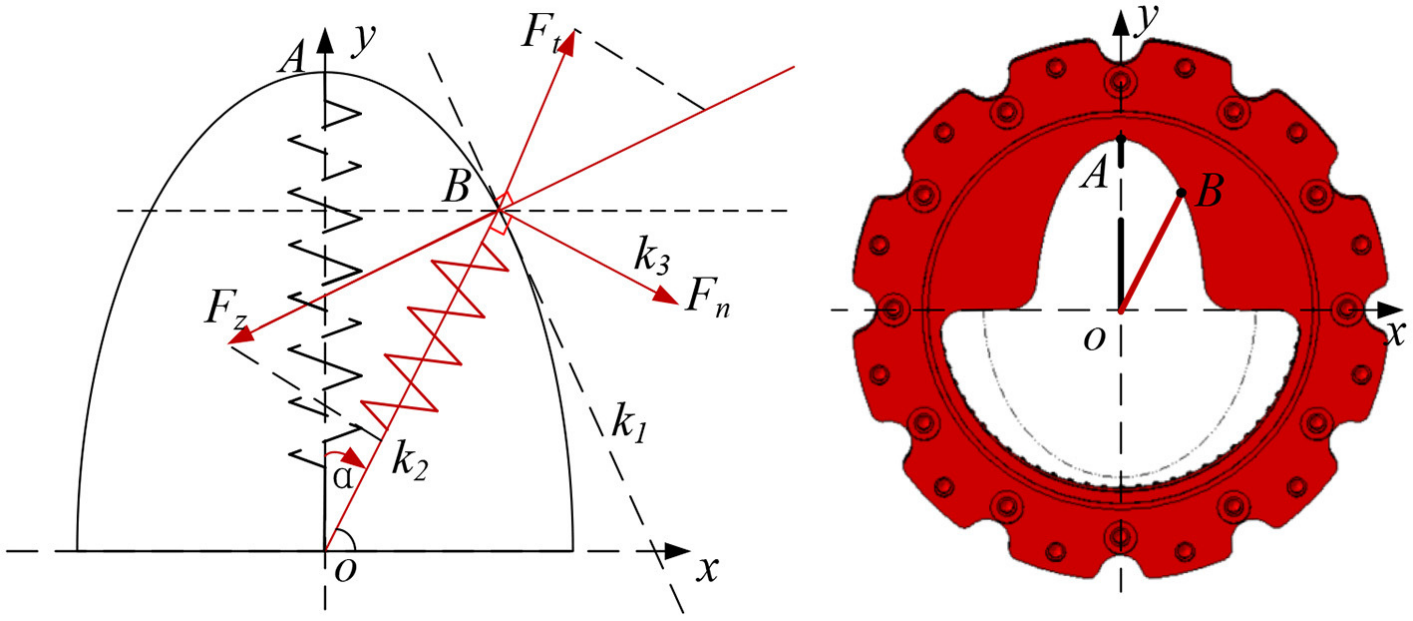
Theoretical analysis of special surface.

Here, for the convenience of analysis, the output flange in Figure 3A is fixed, and the cam follower is idealized as a point. As shown in Figure 4, the cam contour is designed as an ellipse, the center of the cam disc is the o origin, the long axis of the cam contour is in the positive direction of the y axis, and the short axis is in the positive direction of the x axis. The xoy coordinate system is established.

Assuming that the length of the short axis of the ellipse is a and the length of the long axis of the ellipse is b, combined with Figure 4, the general equation of the elliptic curve is parameterized:


(1) $$\begin{array}{l} \{\begin{array}{c} x_{0}=a\cdot \sin\left(\alpha \right)\\ y_{0}=b\cdot \sin\left(\alpha \right) \end{array} \end{array}$$

According to the previous section, the input flange rotates α rad, spring OA also rotates α rad and reach to OB position. At this time, the tangent slope *k*_1_ of the ellipse at point B and the length *c* of OB are:


(2) $$\begin{array}{l} k_{1}=\frac{y_{0}}{x_{0}} \end{array}$$

(3) $$\begin{array}{l} c=\vec{OB}=\sqrt{{x_{0}}^{2}+{y_{0}}^{2}} \end{array}$$

At point B, due to the compression of the spring, the elastic force *F*_*t*_ is generated, then *F*_*t*_ is:


(4) $$\begin{array}{l} F_{t}=2^{*}kk^{*}\left(b-c+l_{0}\right) \end{array}$$

In equation (4), l0 is the initial compression of the spring, and kk is the spring stiffness coefficient.

The slope k2 of Ft can be obtained:


(5) $$\begin{array}{l} k_{2}=-\frac{b^{2*}x_{0}}{a^{2*}y_{0}} \end{array}$$

According to equations (2) and (5), the slope *k*_3_ of the tangential force *F*_*n*_ of the output flange can be obtained as:


(6) $$\begin{array}{l} k_{3}=\left|\frac{k_{1}-k_{2}}{1+k_{1}^{*}k_{2}}\right| \end{array}$$

According to equations (2)–(6), the functional relationship between the output torque *T*_*n*_, deformation angle of the variable stiffness joint α and the initial compression of the spring *l*_0_ can be obtained.


(7) $$\begin{array}{l} T_{n}=f\left(\alpha ,l_{0}\right)=\frac{2*kk*\left(b-\sqrt{{x_{0}}^{2}+{y_{0}}^{2}}+l_{0}\right)}{\left|\frac{k_{1}-k_{2}}{1+k_{1}*k_{2}}\right|}*\\ \;\sqrt{{x_{0}}^{2}+{y_{0}}^{2}}*10^{-3} \end{array}$$

In equation (7), *x*_0_ and *y*_0_ are functions of α, and it can be measured by the encoder.

The expression of static stiffness of variable stiffness joint is:


(8) $$\begin{array}{l} K_{s}=\frac{dT_{n}}{d\alpha } \end{array}$$

**Figure 5:**
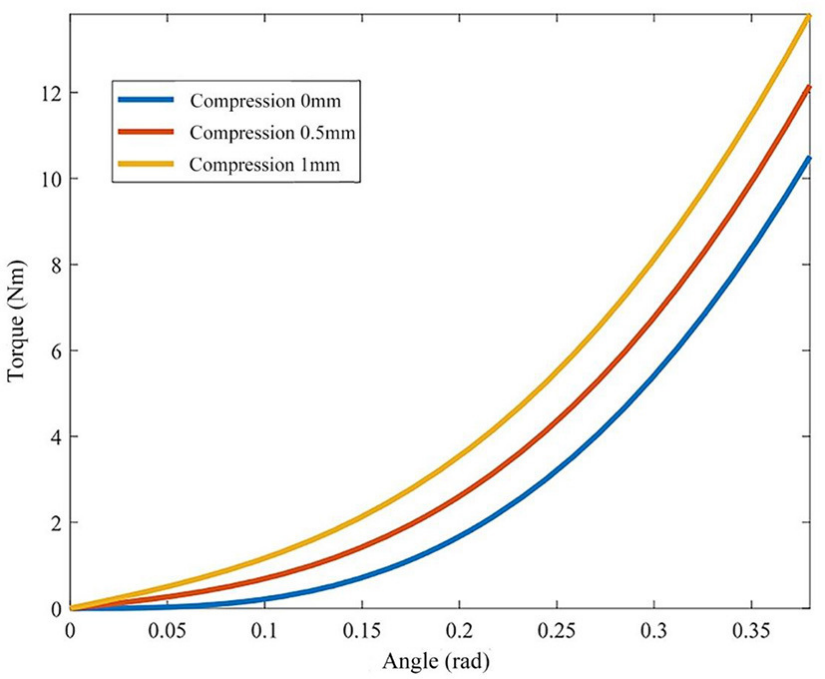
Relationship between joint torque and deformation angle.

According to equation (7), the cam profile on the output flange determines the nonlinear relationship between the output torque *T*_*n*_, the deformation angle of the variable stiffness module α and the initial spring compression *l*_0_, which determines the stiffness characteristics of the joint. The initial compression *l*_0_ of the spring can be changed by the screw actuated by the stiffness adjustment motor to adjust the joint stiffness. Take the spring stiffness coefficient *kk* = 88 N/mm, the ellipse long axis *a* as 60 mm, and the short axis *b* as 30 mm. According to the above theoretical equation, the relationship curve between the output torque *T*_*n*_, the deformation angle α and the spring compression *l*_0_(0, 0.5, and 1 mm) is shown in Figure 5.

It can be seen from Figure 5 that the joint output torque curve shows an upward trend as a whole. With the increase of deformation angle, the joint output torque curve shows an upward trend. The rising slope of the first half is small, the joint stiffness is low and the range of stiffness change is small, so it can be used for resistance training. The second half of the output torque curve has a large rising slope, high joint stiffness and a wide range of stiffness changes, which is suitable for passive training. The greater the initial compression of the spring *l*_0_, the greater the joint stiffness at the same joint deformation angle α. Finally, it is found that the range of joint deformation angle is 0–0.4 rad, the corresponding output torque range is 0–14 Nm, and the stiffness variation range is 0–35 Nm/rad, which meets the requirements of human elbow joint stiffness and torque output, ant it can used in elbow rehabilitation.

## Dynamic modeling and control

### Dynamic model

**Figure 6:**
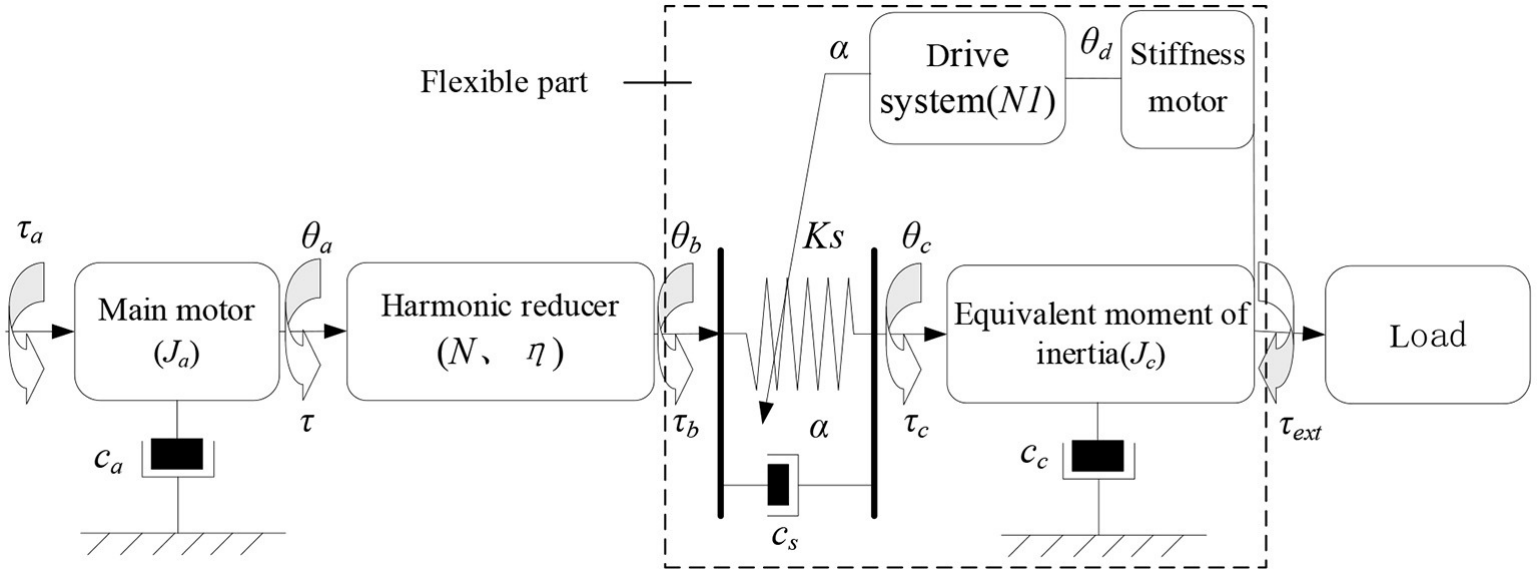
Dynamic model of elbow joint with variable stiffness.

In this section, a variable stiffness joint dynamic model is established to derive the joint transfer function. As shown in Figure 6, the variable stiffness joint is composed of motor, reducer, elastic element, transmission system and load. The main motor provides the power source, which is output to the flexible link through the harmonic reducer. The torque is output from the end of the flexible link and connected with the external load.

The torque of main motor τ_*a*_ satisfy the following relation:


(9) $$\begin{array}{l} \tau_{a}=J_{a}{\ddot{\theta }}_{a}+C_{a}{\overset{\cdot }{\theta }}_{a}+\tau \end{array}$$

In equation (9), *J*_*a*_ and *C*_*a*_ are the inertia and damping coefficient of the main motor, τ is the output torque of the main motor, θ_*a*_ is the output angle of the main motor. Torque and position at the output end of harmonic reducer are τ_*b*_ and θ_*b*_. τ_*a*_ and θ_*a*_ satisfy the following relation,


(10) $$\begin{array}{l} \{\begin{array}{c} \theta_{a}=\frac{\theta_{b}}{N}\\ \tau_{b}=\frac{\tau_{a}}{N_{\eta }} \end{array} \end{array}$$

In equation (10), *N* is the harmonic reduction ratio, η is the transmission efficiency. In this joint mechanism, the flexible link only plays the role of transmitting torque, so the torque at both ends of the flexible link has the following relationship:


(11) $$\begin{array}{l} \tau_{a}=\tau_{c}=J_{c}{\ddot{\theta }}_{c}+C_{c}{\overset{\cdot }{\theta }}_{c}+\tau_{ext}=K_{s}\left(\theta_{b}-\theta_{c}\right)\\ \;+C_{s}\left(\theta_{b}-\theta_{c}\right) \end{array}$$

In equation (11), τ_*c*_is the output torque of the flexible link, *J*_*c*_ is the equivalent inertia of the transmission system, *C*_*c*_ is the damping coefficient of the transmission system, *C*_*s*_ is the damping coefficient of the flexible element, θ_*c*_ is the rotation angle of the output end, τ_*ext*_ is the external load torque. Finally, the dynamic model of variable stiffness joint can be obtained as:


(12) $$\begin{array}{l} \{\begin{array}{c} T_{a}=J_{a}{\ddot{\theta }}_{a}+C_{a}{\overset{\cdot }{\theta }}_{a}+\frac{\tau_{b}}{N_{\eta }}\\ \tau_{b}=\tau_{c}=K_{s}\left(\frac{\theta_{a}}{N}-\theta_{c}\right)+C_{s}\left(\frac{{\overset{\cdot }{\theta }}_{a}}{N}-{\overset{\cdot }{\theta }}_{c}\right)\\ \tau_{c}=J_{c}{\ddot{\theta }}_{c}+C_{c}{\overset{\cdot }{\theta }}_{c}+\tau_{ext} \end{array} \end{array}$$

Then the variable stiffness joint transfer function is:


(13) $$\begin{array}{l} G\left(s\right)=\frac{\tau_{c}\left(s\right)}{\tau_{a}\left(s\right)}=\frac{J_{c}s^{2}\theta_{c}\left(s\right)+C_{c}s\theta_{c}\left(s\right)+\tau_{ext}\left(s\right)}{J_{c}s^{2}\theta_{c}\left(s\right)+C_{a}s\theta_{a}\left(s\right)+\frac{\tau_{c}\left(s\right)}{N_{\eta }}} \end{array}$$

### PID torque control based on bp neural network

Generally, the traditional PID control method is adopted for the joint torque control, and the control command is τ_*d*_. τ_*d*_, τ_*a*_ and τ_*c*_ has the relationship as following,


(14) $$\begin{array}{l} \tau_{a}=k_{p}\left(\tau_{d}-\tau_{c}\right)+k_{i}\int \left(\tau_{d}-\tau_{c}\right)dt+k_{d}\frac{d\left(\tau_{d}-\tau_{c}\right)}{dt} \end{array}$$

In equation (14), *k*_*p*_, *k*_*i*_ and *k*_*d*_ are proportional, integral and differential control parameters, respectively. At this time, the joint torque transfer function is:


(15) $$\begin{array}{l} G_{1}\left(s\right)=\frac{\left(k_{d}s^{2}+k_{p}s+k_{i}\right)G\left(s\right)}{\left(k_{d}s^{2}+k_{p}s+k_{i}\right)G\left(s\right)+s} \end{array}$$

**Figure 7:**
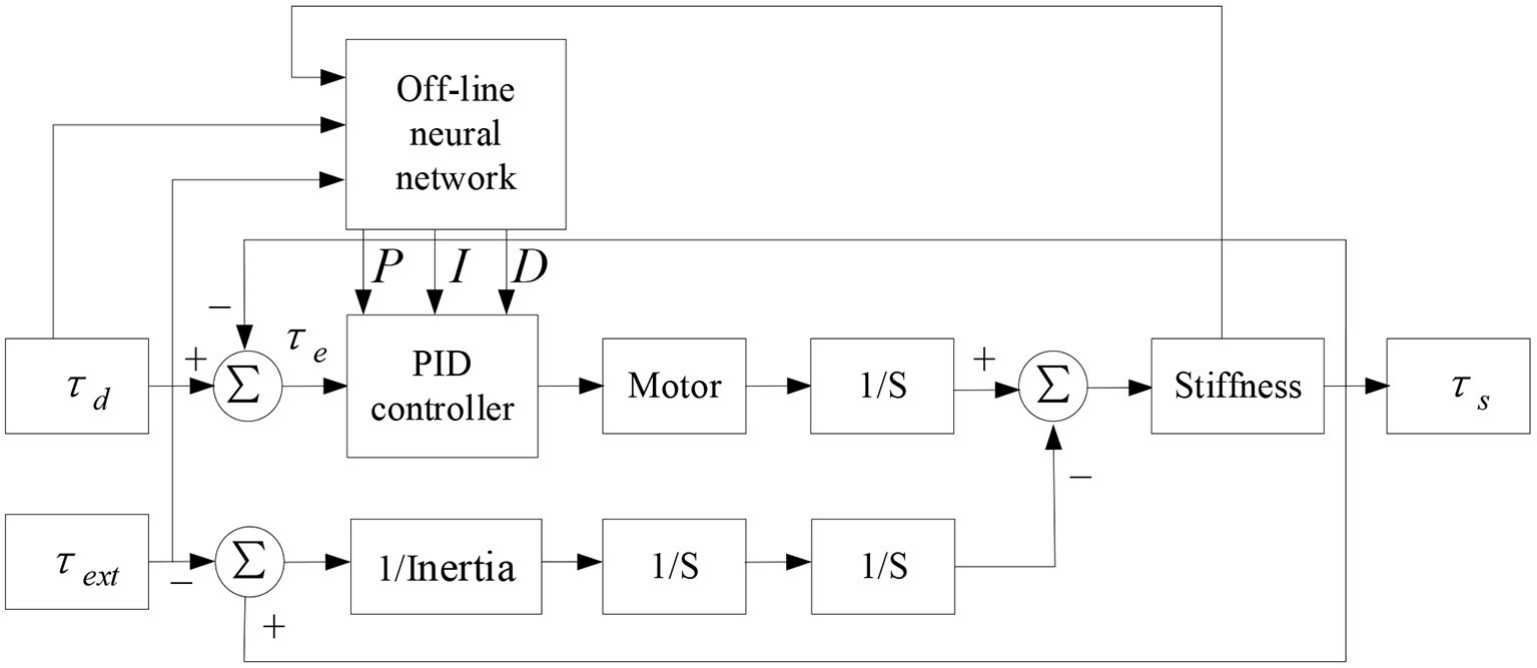
PID control model based on BP neural network.

From equations (12) and (13), it can be seen that the torque response of variable stiffness joint is related to joint stiffness *K*_*s*_, external load τ_*ext*_, torque command τ_*d*_ and PID gain parameters. *K*_*s*_ and τ_*d*_ are determined by the control command, and the external load τ_*ext*_ is determined by the human-machine interaction torque. When the traditional PID control method is adopted, the fixed PID gain parameters are difficult to meet the torque control performance under different stiffness conditions, especially in the case of low stiffness, improper parameter adjustment will produce oscillation, slow response, large overshoot, long system stability time and other phenomena, which will reduce the safety of in rehabilitation training. Therefore, a PID control strategy based on BP neural network is designed as shown in Figure 7.

**Figure 8:**
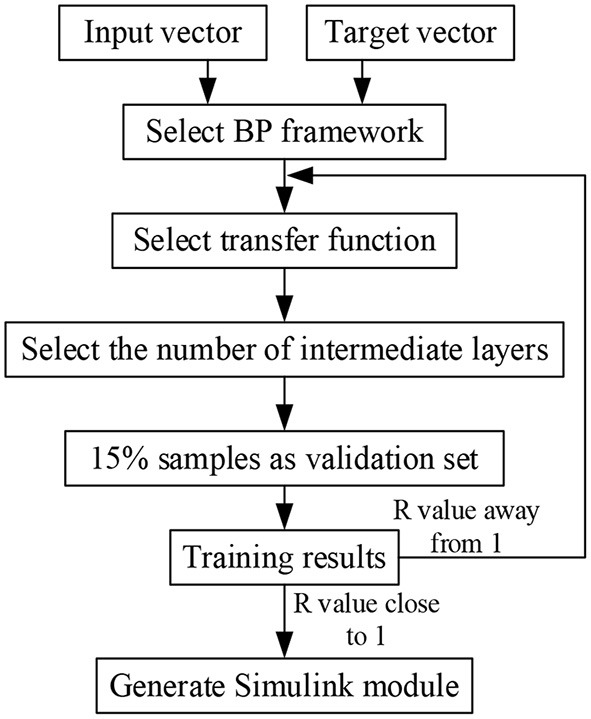
BP neural network construction process.

BP off-line neural network can optimize the gain parameters of PID controller for three groups of variables: different joint stiffness *K*_*s*_, external load τ_*ext*_ and driving torque τ_*d*_, so as to improve the real-time, accuracy and stability of torque response of joints under low stiffness furtherly. The specific implementation process is shown in Figure 8. Select a limited sample set (*K*_*s*_, τ_*d*_, τ_*ext*_) as the input vector, the *K*_*s*_ parameter covers the range of 3–40 Nm/rad, the τ_*d*_ parameter covers the range of 0–20 Nm, and the τ_*ext*_ parameter covers the range of 0–20 Nm/rad. In the case of finite set, the optimal *k*_*p*_, *k*_*i*_, *k*_*d*_ parameters are used as the objective vector. Total 600 groups of samples (Combination 1: τ_*d*_ and τ_*ext*_ remain unchanged, *K*_*s*_ changes, and the optimized *k*_*p*_, *k*_*i*_ and *k*_*d*_ parameters are obtained by simulation. Combination 2: *K*_*s*_and τ_*d*_ remain unchanged, τ_*ext*_changes, and the optimized *k*_*p*_, *k*_*i*_ and *k*_*d*_ parameters are obtained by simulation. Combination 3: *K*_*s*_ and τ_*ext*_ remain unchanged, τ_*d*_ changes, and the optimized *k*_*p*_, *k*_*i*_ and *k*_*d*_ parameters are obtained by simulation). Optimized parameters are shown in Table 1. Due to space limitations, only some parameters are shown here. Because BP neural network has the characteristic of back propagation, which can improve the accuracy of target vector, this paper selects BP neural network framework, its hidden layer adopts tansig transfer function, and its output layer adopts purelin transfer function. The transfer equation between different layers has the following relationship:


(16) $$\begin{array}{l} \{\begin{array}{c} a_{1}=tansig\left(IW^{1,1}\cdot p_{1}+b_{1}\right)\\ a_{2}=purelin\left(LW^{2,1}\cdot a_{1}+b_{2}\right) \end{array} \end{array}$$

**Table 1:** Partial input vector and target vector parameters.

**Input vector**			**Target vector**		
***K_*s*_*(Nm/rad)**	***τ_*d*_*(Nm)**	***τ_*ext*_*(Nm)**	***k_*p*_***	***ki***	***k_*d*_***
3	10	5	2,862.667	42,101.83	6.594912
6	10	5	2,425.731	54,381.63	3.2941
9	10	5	3,103.1	133,517.8	2.352503
12	10	5	2,092.687	75,721.37	0.289952
15	10	5	1,648.609	52,694.09	1.162397
18	10	5	1,712.596	65,955.46	0.892257
21	10	5	1,489.212	54,583.03	0.701764
24	10	5	1,235.035	40,014.22	0.580256
27	10	5	1,134.435	36,022.09	0.477105
30	10	5	1,005.529	34,946.03	0.734407
35	10	5	980.4292	39,073.43	0.66269

**Figure 9:**
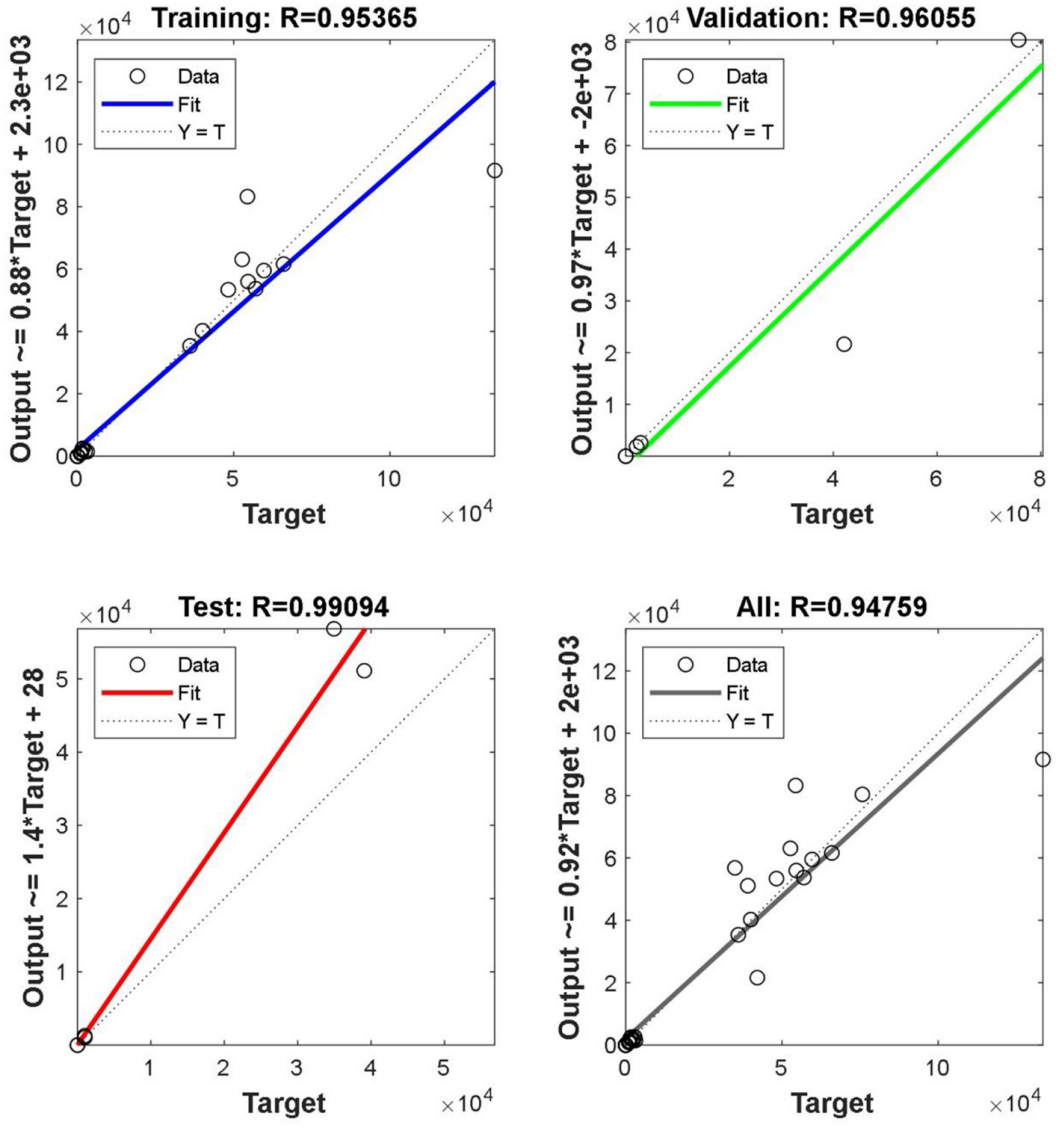
Correlation coefficient of each sample set.

In equation (16), *a*_1_ is the output of the first hidden layer, *a*_2_ is the output of the first output layer, *p*_1_ is the input vector of the first layer, *IW*^1, 1^ is the weight of the first hidden layer, *b*_1_ is the threshold of the first hidden layer, *LW*^2, 1^ is the weight of the first output layer, *b*_2_ is the threshold of the first output layer, 80% of the random samples are used as the training set, 10% of the samples are used as the verification set, and 10% of the samples are used as the test set for training. As shown in Figure 9, the abscissa represents the target output, and the ordinate represents the fitting function between the prediction output and the target output. In Figure 9, the regression r value of the training set is 0.90598, the regression r value of the verification set is 0.99356, the regression r value of the test set is 0.87803, and the overall R value is 0.91155. When the regression r value after training is close to 1, it indicates that the error is very small.

**Figure 10:**
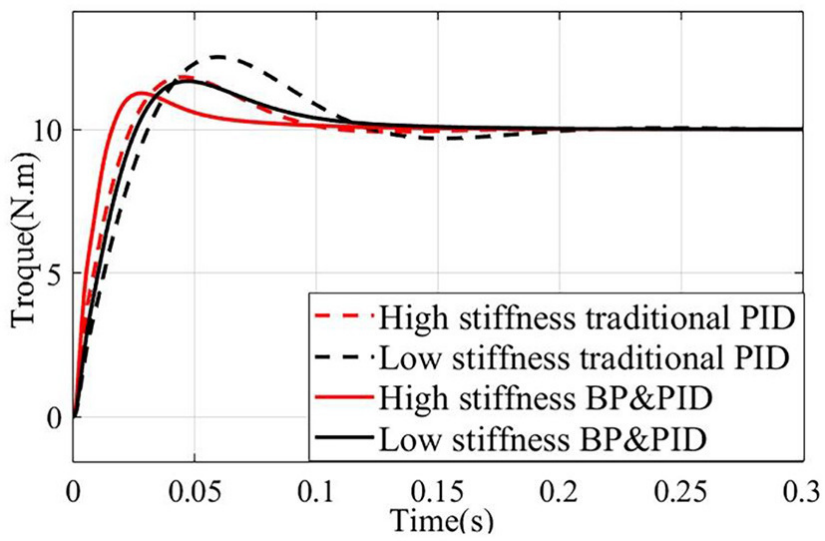
Joint torque response based on PID controller and Gain-scheduled control.

Finally, the PID torque control model based on BP neural network is built in Simulink for simulation analysis. Under the conditions of low stiffness (spring compression of 0 mm) and high stiffness (spring compression of 1 mm), the simulation results are compared with the traditional PID torque control, and the results are shown in Figure 10. It can be seen that the response of the joint based on BP neural network PID torque control is better than that of the traditional PID torque control in the case of low stiffness and high stiffness, especially in the case of low stiffness, which is more suitable for the resistance training of the joint in the case of low stiffness. Under the condition of high stiffness, the joint system based on BP neural network PID torque control reaches the maximum overshoot of 11.6 Nm after 0.03 s, which is 0.02 s faster than the traditional PID torque control and the overshoot is 0.6 Nm smaller. In the case of low stiffness, the joint system based on BP neural network PID torque control reaches the maximum overshoot of 11.8 Nm after 0.045 s, which is 0.02 s faster than the traditional PID torque control, and the overshoot is about 1 Nm smaller.

## Experimental verification

### Experimental platform

**Figure 11:**
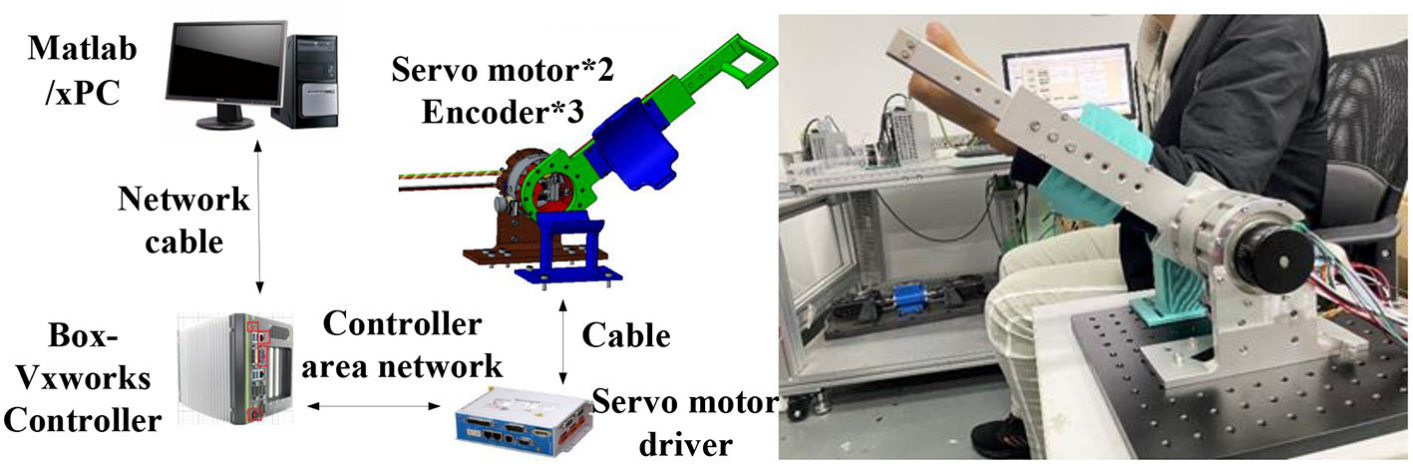
Principle of variable stiffness joint experimental platform.

In the experiment, the simulation platform developed by Links company is used, as shown in Figure 11. On this platform, the static stiffness experiment is carried out firstly, and then the BP neural network PID torque control experiment is carried out to verify the joint torque control performance under the condition of low stiffness. Finally, the isotonic centripetal resistance rehabilitation training experiment is carried out.

The host-computer in the experimental platform runs RT-sim and MATLAB/Simulink software. RT-sim software compiles the Simulink model into C code that can be executed under VxWorks system, and the host-computer is equipped with operation and monitoring modules. The slave-computer is composed of VxWorks, servo motor driver. VxWorks runs the program compiled by RT-sim, and sends the command to the motor servo driver to control the variable stiffness joint. At the same time, the sensor signals in the variable stiffness joint are collected to monitor the joint state.

### Static stiffness test

**Figure 12:**
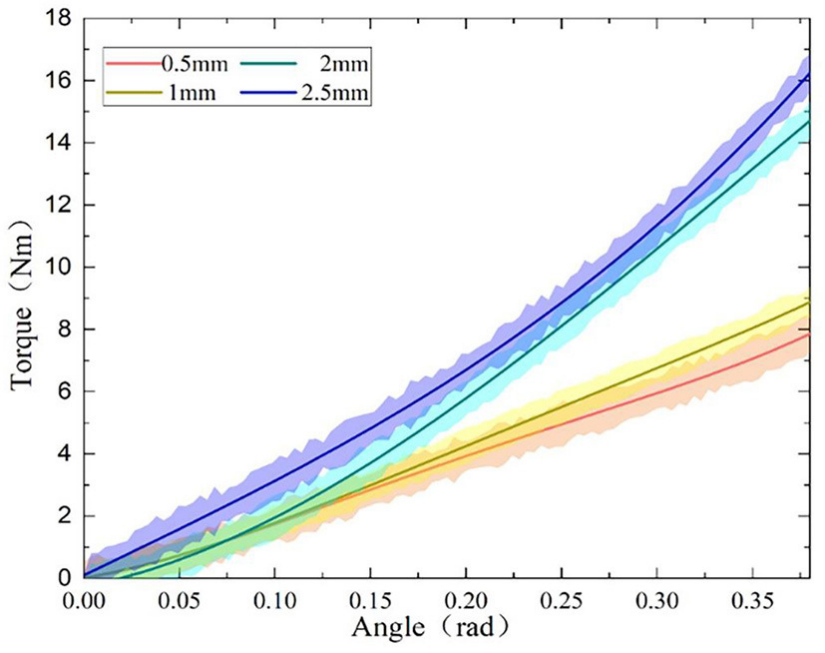
Torque-deformation angle curve.

Firstly, the static torque of the variable stiffness joint is measured. When the joint is fixed, pull the rotating arm through the tension meter to rotate. The length of the rotating arm is 30 cm, and the joint deformation angle is measured by the rotary encoder. Under different initial spring compression, the joint rotates evenly between 0 and 0.35 rad by the same interval, and the measured tension value is recorded to obtain the variable stiffness joint torque. As the deformation angle increases, the joint torque shows an upward trend, and under the same torsion angle, the greater the spring compression, the greater the torque, and the greater the joint stiffness. Compared with the theoretical value in Figure 5, the experimental value in Figure 12 has a large error, which is mainly caused by the machining and assembly errors of the mechanism.

### Torque control experiments

The torque control experiment was carried out under the conditions of low stiffness (spring compression of 0 mm) and high stiffness (spring compression of 1 mm). PID control and BP neural network based PID controller were used to carry out the step response experiment, and the step command was 3 Nm.

**Figure 13:**
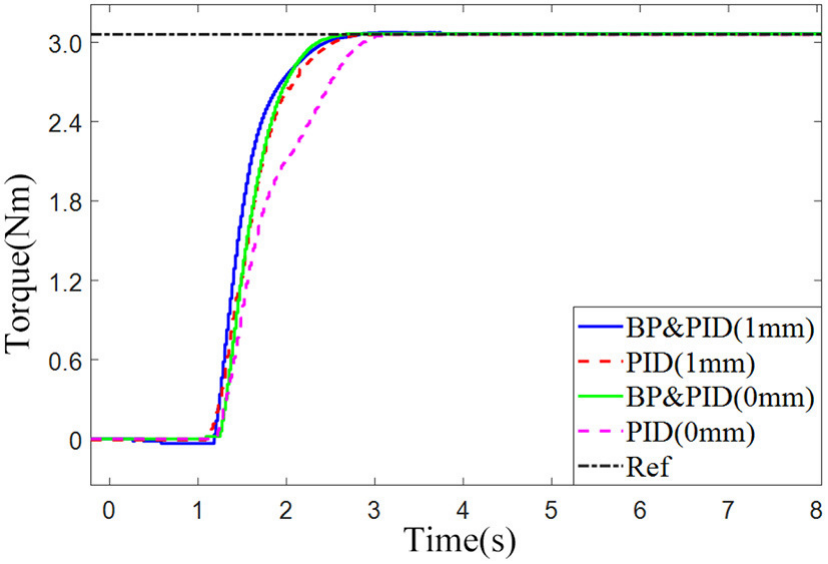
Comparison of joint torque response using different control methods.

As shown in Figure 13, in the low stiffness experimental group, the time for BP neural network PID torque control to reach the steady state is about 1.0 s, and the response time of traditional PID controller is about 1.5 s, an increase of 0.5 s. In the high stiffness experimental group, the time for the BP neural network PID torque control response to reach the steady state is about 1.2 s, the time for the traditional PID response to reach the steady state is about 1.4 s, and the response rate is increased by 0.2 S. It is found that the lower the stiffness is, the more significant the effect of BP neural network PID torque control on improving torque response is, which is more conducive to resistance training.

### Isotonic centripetal resistance training experiment

Finally, the isometric resistance training experiment is carried out. The tester needs to overcome the resistance moment of the variable stiffness joint to complete the elbow flexion movement, and the extension movement is brought back to the initial position by the active torque provided by the variable stiffness mechanism, so as to complete the isometric centripetal resistance training by reciprocating motion. The experiment was divided into two groups. One group set the variable stiffness joint to provide a constant resistance torque of 8 Nm under the condition of high stiffness (spring compression 1 mm) and the other group under the condition of low stiffness (spring compression 0 mm).

**Figure 14:**
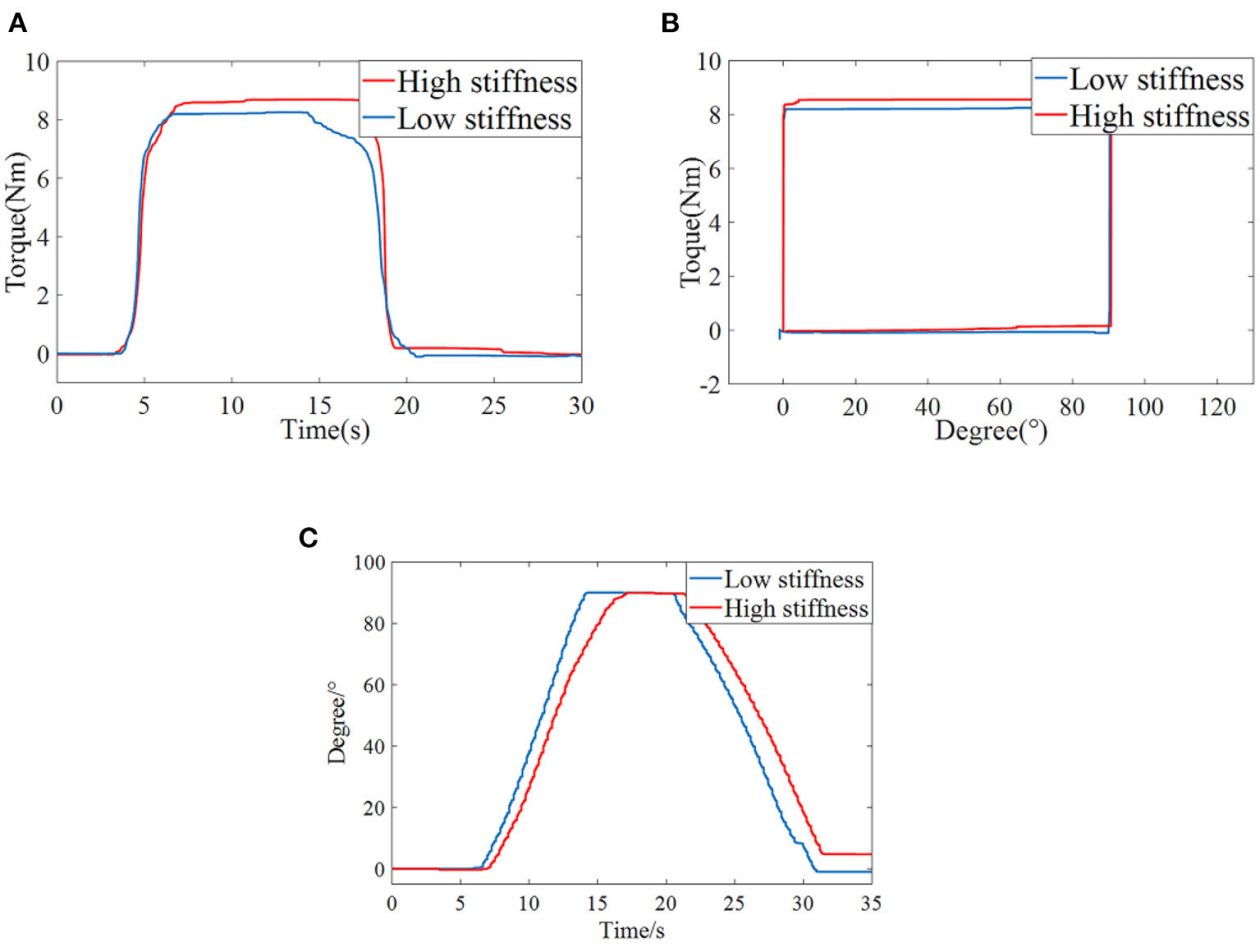
Elbow isotonic centripetal resistance training **(A)** Torque-Time curve **(B)** Torque-Angle curve **(C)** Angle-Time curve.

Figure 14A shows the torque-time curve of isometric concentric resistance training. 0–4s is the preparation stage for the tester, and 4–20s is the active resistance training stage. The human-machine interaction torque gradually rises from 0 to 8 Nm within about 1s and maintains for a period of time. 20–30s is the relaxation stage. The human elbow joint reaches the limit flexion position, and the elbow joint starts to extend driven by the variable stiffness joint. The human-machine interaction torque gradually decreases from 8 to 0 Nm. The torque-angle curve is shown in Figure 14B. Due to the active torque of the user's elbow joint, the human-machine interaction torque rises sharply at 0° until the mechanism starts to rotate when it overcomes the set isotonic resistance torque of 8 Nm, and the rotation angle moves from 0 to 90°. At this time, the elbow joint flexes to the limit position and is about to enter the relaxation stage. After that, the human elbow joint release active torque, and the human-machine interaction torque drops to 0 Nm at 90° rapidly. Then the variable stiffness joint drives the elbow to extend, and the joint angle returns to 0 from90. The angle-time curve in the training process is shown in Figure 14C, corresponding to the preparation stage, the mechanism is in the zero position and does not rotate. In the resistance training stage, the mechanism keeps moving at a constant speed, from 0 to 90°. If the user maintains the torque, the rotation angle of the variable stiffness joint does not change. In the relaxation stage, the mechanism drives the patient's elbow joint to return to its original position. It can be seen from the Figure that under high and low stiffness, the flexible elbow rehabilitation robot can complete isometric resistance training, and the torque is stable, which verifies that the PID torque control method based on BP neural network designed in this paper is suitable for torque control ability under different stiffness again.

## Conclusions

The driving joint of the traditional upper limb rehabilitation robot is rigid, which is difficult to ensure the safety and comfort of the rehabilitation process. To solve this problem, this paper proposes a variable stiffness joint applied to the elbow rehabilitation robot. The joint adopts the variable stiffness principle of special curved surface, and the trapezoidal screw in the joint has a self-locking function, and the stiffness can be maintained without the continuous output torque of the stiffness adjustment motor. In the control aspect, the BP neural network based PID controller is used to improve the torque control response performance by adjusting the gain parameters under different stiffness. The experimental platform of elbow rehabilitation robot with variable stiffness joint is built, and the isotonic centripetal resistance training experiment of elbow joint is carried out to verify the effect of BP neural network PID torque control. The future work will be combined with online neural network to match the elbow stiffness of patients in real time for resistance rehabilitation training.

## Data availability statement

The original contributions presented in the study are included in the article/supplementary material, further inquiries can be directed to the corresponding author/s.

## Author contributions

BH: conceptualization, supervision, and project administration. BM: writing-original draft and visualization. SL: methodology and formal analysis. HY: supervision and project administration. All authors contributed to the article and approved the submitted version.

## Funding

This work was supported in part by National Key R&D Program of China (2020YFC2005800 and 2020YFC2005804), Natural Science Foundation of Shanghai (20ZR1437800), and Biomedical Science and Technology support project of Shanghai (22S31901400).

## Conflict of interest

The authors declare that the research was conducted in the absence of any commercial or financial relationships that could be construed as a potential conflict of interest.

## Publisher's note

All claims expressed in this article are solely those of the authors and do not necessarily represent those of their affiliated organizations, or those of the publisher, the editors and the reviewers. Any product that may be evaluated in this article, or claim that may be made by its manufacturer, is not guaranteed or endorsed by the publisher.
